# White-Light Spectral Interferometry for Characterizing Inhomogeneity in Solutions and Nanocolloids

**DOI:** 10.1021/acsnanoscienceau.2c00014

**Published:** 2022-07-18

**Authors:** Aparna Praturi, Stefan Schrod, Bhanu Pratap Singh, Parinda Vasa

**Affiliations:** † Department of Physics, Indian Institute of Technology Bombay, 400076 Mumbai, India; ‡ Department of Physics, University of Regensburg, 93053 Regensburg, Germany

**Keywords:** spectral interference, spectral broadening, inhomogeneous broadening, Michelson interferometer, Lorentz−Drude model, material dispersion, metal nanoparticles

## Abstract

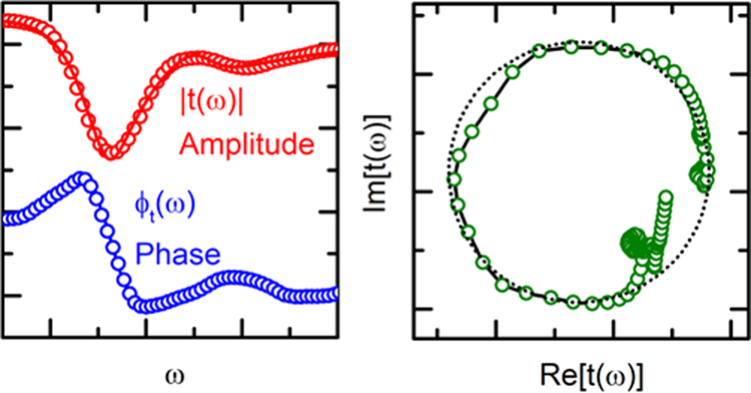

We demonstrate the use of white-light spectral interferometry as an effective technique involving only linear optical interactions and a partially coherent light source to measure the complex transmission response function of optical resonance and to determine the corresponding variation in the refractive index relative to a reference. We also discuss experimental arrangements to increase the accuracy and sensitivity of the technique. The superiority of the technique over single-beam absorption measurements is demonstrated by the accurate determination of the response function of the chlorophyll-*a* solution. The technique is then applied to chlorophyll-*a* solutions of varying concentrations and gold nanocolloids to characterize inhomogeneous broadening. Results on the inhomogeneity of gold nanocolloids are also supported by transmission electron micrographs, showing distributions of the size and shape of the constituent gold nanorods.

## Introduction

Though very basic, the Lorentz–Drude or Lorentz model for the optical response satisfactorily describes absorption spectra associated with a wide variety of material resonances. A limitation of this model is that damping has been introduced phenomenologically.^1,2^ It offers an effective spectral width without distinguishing between different damping mechanisms, like population relaxation, polarization dephasing, and spectral broadening due to other coherent and incoherent interactions. As a result, the line shape of the optical spectra measured from an ensemble of emitters usually deviates from the homogeneously broadened line shape observed in a spectrum of an individual quantum emitter.^1,2^ Such line shape modifications are also a result of variations in size, shape, or composition of the emitter molecules within the ensemble, giving rise to the distribution in their optical resonance frequency, oscillator strength, and/or dephasing time.^3−5^ Another well-reported cause of line shape modification is fluctuations in the local environment of each emitter, resulting in quasistatic disorder and consequently in an inhomogeneous broadening of the optical spectra.^6,7^ Artificial nanostructures such as quantum dots and metal nanoparticles show particularly pronounced inhomogeneous broadening in their line shapes^8^ as current synthesis or nanofabrication techniques generally do not provide completely identical emitters and cannot offer full control over the optical properties of each individual nanostructure. Similar disorder effects also strongly influence the optical spectra of inorganic and organic semiconductors and molecular aggregates. Several optical techniques have been developed over the years to analyze optical line shapes of individual emitters as well as their ensembles. Of these, single emitter spectroscopy^9−11^ can directly determine the homogeneous line shape of an individual emitter; but in an ensemble, inhomogeneous broadening has a significant contribution to the effective line shape. Various nonlinear optical techniques such as hole-burning spectroscopy^1,12,13^ and impulsive four-wave mixing techniques like transient grating or photon echo spectroscopies^14−16^ have been explored to distinguish between homogeneous and inhomogeneous line shape broadening in an ensemble. More recently, two-dimensional (2D) femtosecond optical spectroscopy in the visible spectral range has rapidly become increasingly important in probing dephasing dynamics and, more generally, the dynamics of optical excitations in nanostructures.^2,17−20^ Nevertheless, femtosecond time-resolved 2D optical measurements are rather challenging, requiring sophisticated ultrafast lasers even though several elegant designs have been developed^21−23^ that help in reducing the complexity of these powerful nonlinear experiments. Moreover, the quantitative interpretation of some of these measurement techniques is currently being actively discussed.^24,25^

White-light spectral interferometry (WLSI) is an interferometric technique in which a partially coherent broad-band light source is used to illuminate an interferometer, and the resulting interferogram is spectrally resolved and recorded. WLSI has found many applications in measuring distances, thicknesses or displacements^26,27^ and material dispersion;^28,29^ it is also used in spectrometer calibration,^30−32^ optical profilometry,^33,34^ broad-band optical communication applications,^35^ and to investigate dynamics.^36^ In this work, we use WLSI to measure the relative phase accumulated by a beam by passing through a material exhibiting optical resonance with respect to a transparent reference. We further use it to determine the extent of deviation of the optical response from the Lorentz model in chlorophyll-*a* solutions of varying concentrations and of two types of gold nanocolloids. Though the technique cannot directly distinguish homogeneous and inhomogeneous broadening,^1,37,38^ it can clearly show the deviation from the optical response expected from the Lorentz model that is generally used to describe homogeneous broadening. As the measurements have been made on ensembles, the deviation from Lorentz response is attributed to sample inhomogeneity. In the case of nanocolloids, size/shape inhomogeneity is also confirmed by transmission electron microscopy (TEM) measurements. We also demonstrate that simply fitting of optical response to Lorentz or Voigt line shapes does not conclusively provide information about the alteration in the line shape or the extent of inhomogeneous broadening. With WLSI, a clear indication of line shape deviation can be observed, which can also be quantified. Hence, the technique can find applications in low-cost automated sample characterization and quality control. The WLSI technique also gives a variation in the refractive index of a solvent against the reference sample over a broad range of frequencies. Both the measurements of the refractive index over a wide range of frequencies and the estimation of inhomogeneity are crucial in various applications such as in characterization and identification of materials as well as for signal processing. Several techniques based on interferometry, Abbe refractometry,^39^ ellipsometry,^40^ and the method of minimum deviation^41^ have been employed for the determination of the refractive index. Some of these techniques are extremely sensitive, but they generally provide the value of the refractive index only at a given frequency or set of discrete frequencies and do not provide any estimate of the inhomogeneous broadening. Hence, interpolation or empirical formalism often has to be used in conjunction to obtain the refractive index in a broad spectral band. In WLSI, the phase difference between the reference and the sample can be determined with high sensitivity as the method involves only linear optical interactions. This spectral phase is analyzed further to compare the inhomogeneity of colloids as well as to determine the differential refractive index. Another distinct advantage of WLSI is that a partially coherent broad-band light source like an light-emitting diode (LED) or a lamp is sufficient. It does not require a sophisticated ultrafast laser system as in the case of time-resolved nonlinear techniques, making it highly accessible as well as promising for widespread usage.

In this paper, we use the spectral phase retrieved from WLSI to determine the full complex transmission response function and estimate the inhomogeneous broadening present in the line shapes of an ensemble of both natural (chlorophyll-*a*) and synthesized (gold nanocolloids) ensembles.

## Theory and Background

A beam of light passing through a medium undergoes changes in its amplitude and phase as determined by the complex refractive index *n*(ω) + *i*κ(ω) of the medium. We can denote these changes in terms of a transmission response function of the sample, *t*(ω) = |*t*(ω)|*e*^*i*ϕ_t_(ω)^ whose amplitude is given by |*t*(ω)| = e^–κ(ω)*k*_0_d^ and ϕ_t_(ω) = Δ*n*(ω)*k*_0_*d*. Here, *d* denotes the thickness of the medium and Δ*n* denotes the difference between the refractive index of the medium and that of the surrounding. Correspondingly, if two different samples of the same thickness, *d*_0_, but a small difference in their complex refractive indices represented by *n* + *i*κ and (*n* + δ*n*) + *i*(κ + δκ) are placed in different arms of a Michelson interferometer, the phase difference calculated from the resulting interferogram has the form ϕ(ω) = 2[δ*n*(ω)]*k*_0_*d* + ω(τ_1_ – τ_2_), where the latter term denotes the difference in the path length of the two arms.

Now, if one of the samples has an optical resonance at ω_0_ within the spectral range under observation while the other sample is transparent, then the first term of the interferogram phase ϕ(ω) along with |*t*(ω)| gives the transmission response function of the absorbing sample. The optical response of the sample can be modeled as a complex Lorentz function under the Lorentz oscillator model^42^ as
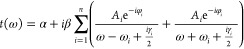
1where α and β are slowly varying background scaling factors, φ_*i*_ represents the relative phases between the resonances, and ω_*i*_ and γ_*i*_ are the position and width (full width at half-maximum, FWHM) of the resonances. Thus, determination of ϕ(ω) enables the measurement of even small variations in refractive indices over a broad spectral range.

**Figure 1:**
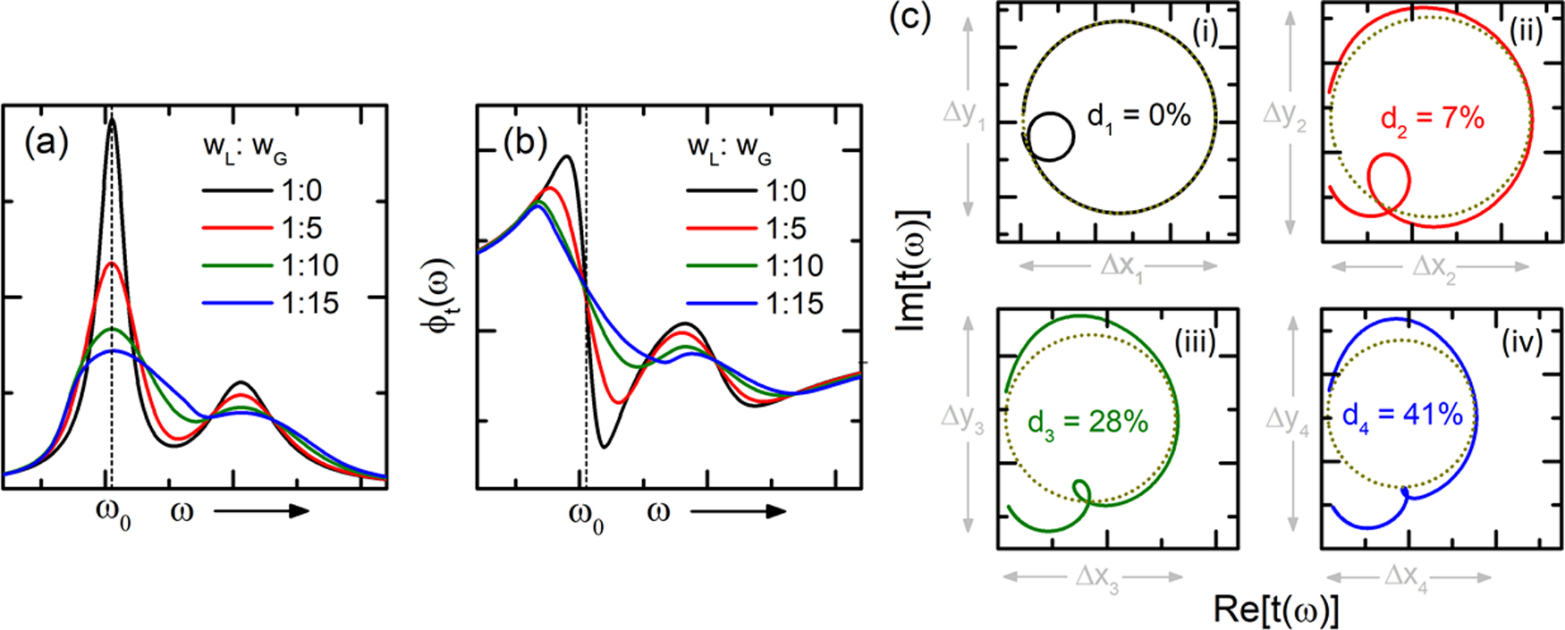
(a) Amplitude |*t*(ω)| and (b) phase ϕ_t_(ω) of the modified transmission response function *t*(ω) for different ratios of the Lorentzian width to the Gaussian widths (*w*_L_/*w*_G_) indicating varying degrees of Gaussian inhomogeneity. (c) Distortion in the circular shape of *t*(ω) plotted on a complex plane for four levels of inhomogeneity. The values of the percentage distortion function *d* for four *w*_L_/*w*_G_ ratios considered here (i) 1:0, (ii) 1:5, (iii) 1:10, and (iv) 1:15 are indicated in the figures.

In eq 1, the damping rate γ is considered to be originating from homogeneous dephasing due to population relaxation and polarization damping. However, for an ensemble of emitters, differences in size, shape, and composition of the emitters can give rise to variations in resonance frequency, oscillator strength, and dephasing time among emitters. Also, variations in the local environment of each emitter, on a time scale exceeding the homogeneous dephasing time, may result in quasistatic disorder and hence an inhomogeneous broadening of the optical spectra.^17−19^ Unfortunately, it is not always possible to estimate the inhomogeneous broadening by analyzing the transmission spectrum alone. However, if complete spectral phase information is available, it is possible to estimate the inhomogeneity. This can be done by plotting the complete transmission response function, *t*(ω), on a complex plane.^42−44^ In the absence of inhomogeneous broadening *t*(ω) on the complex plane is a perfect circle under the Lorentz oscillator model because the real and imaginary parts are in quadrature. Any sort of inhomogeneous broadening distorts the circle. To demonstrate this effect, an example of Gaussian inhomogeneity described by the Gaussian distribution function, , where μ is the mean and σ is the standard deviation of the distribution is discussed. The FWHM of the Gaussian distribution (*w*_G_) is related to the standard deviation σ as . Hence, to include the inhomogeneous broadening effects, we convolute the Lorentzian transmission response function, *t*(ω) consisting of two resonances (main peak at ω_0_) with Gaussian distribution functions with their respective center frequencies located at the Lorentzian resonance positions. The plots showing the resulting amplitude and phase after the convolution for various ratios of the Lorentzian width (*w*_L_ = γ) to the Gaussian width (*w*_G_ = 2.355σ; *w*_L_/*w*_G_ = 1:0, 1:5, 1:10, and 1:15) are shown in Figure 1a,b, respectively. Plotting the real and imaginary parts of the modified response function on the complex plane (Figure 1c) clearly reveals that inhomogeneous broadening distorts the circular shape corresponding to the Lorentzian shape (purely homogeneous broadening). Defining a percentage distortion function , we see that higher inhomogeneity results in higher distortion. Therefore, determination of the full complex response function *t*(ω) not only measures the difference in the refractive indices but also characterizes the degree of inhomogeneity in a given sample.

## Methods

**Figure 2:**
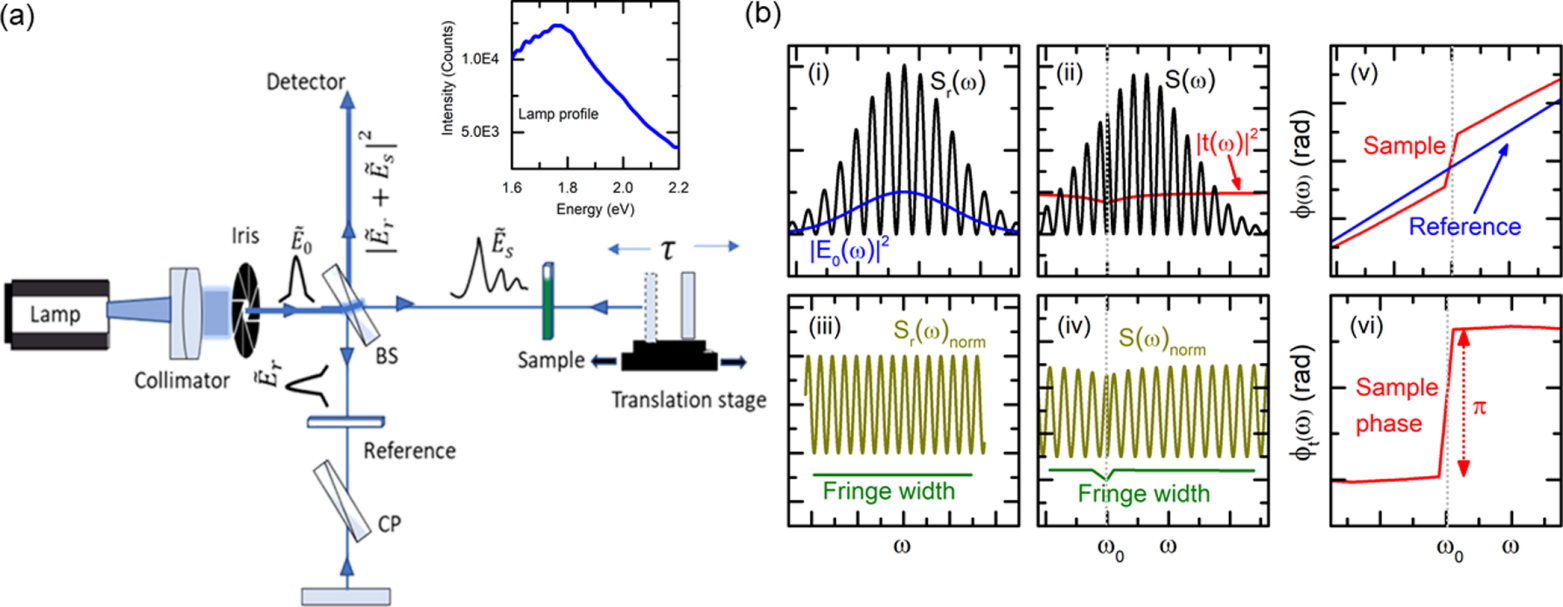
(a) Schematic of the Michelson interferometer used to implement WLSI. BS represents the beam splitter, whereas CP represents the compensator plate. The inset shows the spectrum of the white lamp used as the light source in our experiments. (b) Various steps [(i)–(vi)] in the spectral phase extraction process are shown by simulating a source with Gaussian electric field distribution *Ẽ*_0_(ω) and a sample characterized by a complex Lorentz transmission function, *t*(ω). (i) Source spectrum and interferogram without the sample, (ii) spectrum and interferogram with the sample, (iii) normalized interferogram and fringe width without the sample, (iv) normalized interferogram and fringe width with the sample, (v) retrieved spectral phase with and without the sample, and (vi) sample spectral phase showing the phase jump of π across the resonance.

We use a Michelson interferometer setup shown in Figure 2a to perform white-light spectral interferometry measurements. A temperature-stabilized, unpolarized broad-band beam of light from a tungsten-halogen lamp (Thorlabs) with electric field *Ẽ*_0_(ω) is split by a beam splitter (BS) into a time-delayed reference beam with electric field *Ẽ*_r_(ω) = *E*_0_(ω)e^–*i*ωτ_1_^, where τ_1_ represents the time delay, and a sample beam with modulated electric field *Ẽ*_s_(ω) = *t*(ω)*Ẽ*_0_(ω). For the observation of fringes with good contrast, τ_1_ should be on the scale of the coherence time of the source. A larger delay time reduces the fringe visibility. Here, we have chosen it on the scale of 50–100 fs, which can be adjusted using a manual translation stage with a micrometer screw. The two beams are then recombined at the BS, and the interferogram [*S*_t_(ω)] resulting from the addition of two electric fields, *Ẽ*_t_(ω) = *Ẽ*_r_(ω) + *Ẽ*_s_(ω), is spectrally resolved using a 50 cm focal length spectrometer (Acton Spectra Pro) consisting 150 grooves/mm grating and a CCD camera cooled to −70 °C. The final frequency spectrum thus reads
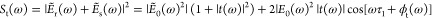
2

The amplitude of the response function can be easily determined by normalizing the sample beam spectrum with the reference beam spectrum. Calculation of the phase of the response function is more intricate and involves determining the fringe widths of the interferogram and subtraction of the linear phase contribution. Due to the configuration of the interferometer, ϕ(ω) here corresponds to a double pass through the sample. The detailed procedure to extract the phase is demonstrated in Figure 2b with the source beam represented as a Gaussian function and the transmission response function *t*(ω) as a complex Lorentz function. Panels (i) and (ii) show a reference interferogram without the sample corresponding to the interference between two Gaussian spectra with time delay τ_1_ and the sample interferogram with the sample inserted in one of the arms, respectively. The numbers of fringes in (i) and (ii) are different due to the difference in time delays in the two cases. The sample is characterized by optical resonance at frequency ω_0_. Both the interferograms are normalized with the incident beam spectrum as shown in panels (iii) and (iv), respectively. The fringe widths of each interferogram as a function of frequency (average of frequencies corresponding to the minimum and maximum amplitudes) are also shown. For the reference interferogram, the fringe width is a constant, whereas for the sample, there is a clear variation in the proximity of the resonance frequency. As adjacent maxima or adjacent minima have a phase difference of 2π, the fringe spacing is converted to the phase by scaling it with 2π. Next, the phase accumulated with an increasing number of maxima is plotted as a function of frequency corresponding to the maxima. Panel (v) shows the phase retrieved from the fringe spacings shown in panels (iii) and (iv). The reference interferogram shows a linearly increasing phase with frequency, whereas that corresponding to the sample shows a phase jump at the resonance frequency. The retrieved phase, ω|τ_1_ – τ_2_| + ϕ_t_(ω), from the fringe pattern (iv) has a linear contribution from the time delay and a dispersive component, i.e., ϕ_t_(ω), or the phase of the response function *t*(ω). The difference in slopes corresponds to the unequal time delays in the two cases. Panel (vi) shows the extracted ϕ_t_(ω) after subtracting the linear term ω|τ_1_ – τ_2_| from the total phase. The ϕ_t_(ω) extracted exhibits the expected π-jump of a complex Lorentz function at the resonance frequency, which is in agreement with the phase obtained from the complex Lorentz function given in eq 1 for a single resonance.

## Results and Discussion

**Figure 3:**
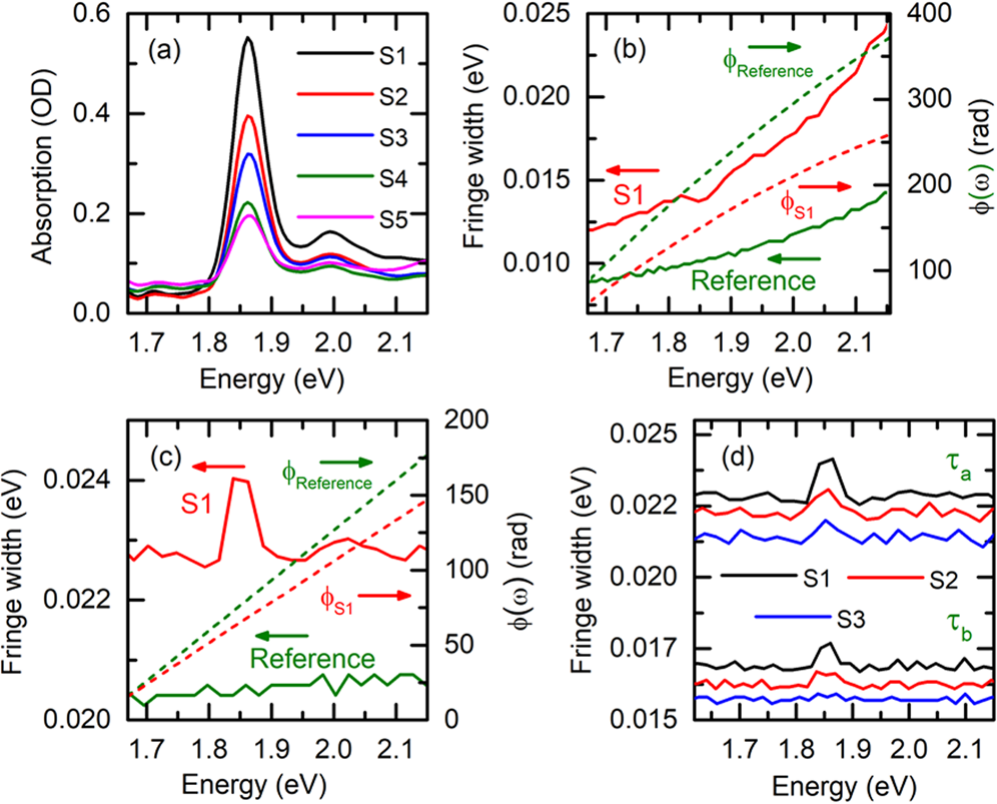
(a) Absorption spectra of the chlorophyll-*a* solutions in ethanol. There is 3.6% fluctuation in the spectral width of the main peak with dilution. Fringe widths and retrieved phase of the recorded reference and sample (solution: S1 with the highest concentration) interferograms (b) without and (c) with the compensator plate. (d) The effect of varying time delays (τ_a_ and τ_b_, τ_a_ < τ_b_) on the S/N ratio of the fringe width data for solutions S1, S2, and S3. It is observed that a lower time delay (τ_a_ corresponding to the higher fringe width) considerably improves the S/N ratio. The S/N for τ_a_ is better compared to τ_b_ by a factor of ∼2 for all three samples. Sample S3 has half the concentration of S1, whereas S5 has one-fourth concentration of S1. S2 and S4 correspond to intermediate concentration.

While experimentally implementing WLSI, two factors playing a key role are the material dispersion introduced by the optical elements of the interferometer and the sensitivity of the setup to detect the smallest change in the refractive index. In the following paragraphs, we address these two aspects before demonstrating the technique to estimate the inhomogeneous broadening. We have used a chlorophyll-*a* solution in ethanol as the test sample. The starting solution concentration (S1) is chosen so that the peak absorption (OD) for a 1 mm path length is ∼0.5. It is then progressively diluted to investigate the effect of concentration on the spectral profile. The chlorophyll-*a* extracted from spinach was procured commercially (Sigma-Aldrich). Its solution in ethanol was taken in a 1 mm path length fused silica cuvette and placed in the sample arm. The absorption spectra of the solutions S1–S5 with decreasing concentration are shown in Figure 3a. All of them show the main absorption peak at 1.85 eV (665 nm) corresponding to its *Q*_*y*_(0 – 0) transmission and a broad shoulder at 2.05 eV (576 nm). The fluctuation (no specific trend with concentration) in the spectral width of the main peak observed while fitting spectra shown in Figure 3a with the Voigt profile is 3.6%. We first discuss ways to address the material dispersion and optimize the accuracy and sensitivity of the technique.

Material dispersion of the optical components leads to an additional frequency-dependent phase, which is manifested as frequency-dependent fringe width even in the absence of the sample. This frequency dependence poses a major obstacle in accurately calculating the phase of the sample transmission response as well as limits the sensitivity of the setup. Figure 3b depicts the fringe width and the phase calculated for the reference (green lines) and the chlorophyll sample (solution: S1, highest concentration, red lines) interferograms without compensating for the material dispersion of the interferometer. The major contribution to the dispersion is from the beam splitter (BS) as the sample beam traverses the BS twice. The frequency-dependent fringe width obtained from the reference interferogram (green solid line) leads to a nonlinear total phase (green dotted line), even without the dispersive sample. In the absence of the sample, the reference phase arises due to the path difference between the two arms and should be linearly dependent on the frequency (eq 2). The corresponding measurements for the sample interferogram show that the curvature of the phase is different from the reference interferograms, but the phase jumps corresponding to the optical resonances are completely masked by the curvature, thus making it difficult to extract the required phase, ϕ_t(ω)_. The expression for the fringe width in the presence of this material dispersion-induced additional frequency-dependent phase ϕ*_a_*(ω) can be obtained by a series expansion given by, i.e., ϕ*_a_*(ω) = ϕ_0_ + ϕ_1_ω + ϕ_2_ω^2^ + ϕ_3_ω^3^ + ....^43^ Here, the curvature of the phase is also sensitive to the time delay, any small changes in the optical path length while introducing the sample also results in incorrect phase retrieval.

An effective way to minimize these effects of material dispersion is to balance the two arms of the interferometer using identical optical components, thereby canceling out the additional phase. In the Michelson interferometer, this also includes compensation for the inherent imbalance between the two arms due to the BS. We place a glass plate made of the same composition and of comparable thickness as the BS as a compensator plate (CP) in the reference arm. The fringe widths and the retrieved phase after inserting the CP are shown in Figure 3c. The reference interferogram now shows a constant fringe width (green solid line), leading to a linear phase (green dotted line). The fringe width of the sample interferogram is also constant except at the resonance positions of the sample, and the total phase does not show any overall curvature making the subtraction of the linear phase to retrieve the desired phase of the transmission response function ϕ_t_(ω) clearly more accurate. The retrieved phase and its relation with the line shape of the absorption peak for samples S1–S5 is discussed below.

In fringe width measurements corresponding to chirp-compensated reference and sample interferograms shown in Figure 3c, there is a considerable amount of noise. This is because the sensitivity of the technique also depends heavily on the signal-to-noise ratio (S/N) of the fringe width determination. In evaluating the fringe widths from the interferogram, noise is not only contributed by the spectrometer but also from the finite memory holding of the analyzing routine used to obtain the fringe widths. Figure 3d shows the S/N ratio of solutions S1–S3 at two different time delays, τ_a_ and τ_b_, τ_a_ < τ_b_. From the figure, it is evident that the S/N ratio is considerably higher when the fringe widths are larger (or the time delay is shorter). The S/N for S1 for τ_a_ is ∼11 (∼5 for τ_b_), and for S3, it is ∼3 (∼1.5 for τ_b_). Nevertheless, the fringe width needs to be smaller compared to the spectral width of the resonance to accommodate at least a few fringes within the spectral width. Hence, adjusting the path length to modify the time delay such that it leads to the widest fringes that can still resolve the resonances is an efficient way of enhancing the S/N ratio. The ratio also depends on the sample concentration.

**Figure 4:**
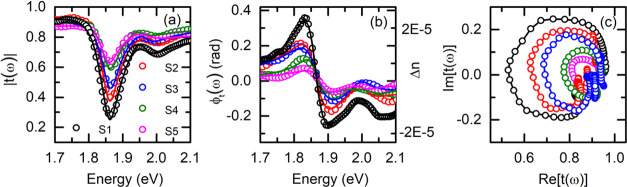
Measured (a) amplitude and (b) phase of the transmission response function *t*(ω) of chlorophyll-*a* solutions S1–S5 in ethanol. Concentrations are the same as shown in Figure 3a. (c) Transmission response function *t*(ω) plotted on the complex plane. The distortion value, *d*, increases with dilution, indicating a small amount of concentration-dependent inhomogeneity, which is not perceptible in |*t*(ω)| shown in (a). In (c), lines are to guide the eye.

The transmission spectra, |*t*(ω)|, of solutions S1–S5 are shown in Figure 4a. The spectra show the major dip at 1.85 eV (665 nm) with an FWHM of 0.044 eV corresponding to the *Q*_*y*_(0 – 0) absorption peak and a broad shoulder at 2.05 eV (576 nm). The spectral width of the main peak on fitting Lorentzian response (eq 1) exhibits a fluctuation of ∼6% similar to that observed in the case of fitting data shown in Figure 3a. The corresponding retrieved phase of the transmission response functions of chlorophyll-*a* solutions (|*t*(ω)| shown in Figure 4a) obtained after eliminating chirp and maximizing the S/N ratio is shown in Figure 4b. The experimentally obtained values (circles) are fitted to eq 1 (lines). For the spectrometer and grating we have used, the spectral resolution in the visible range of the spectrometer is ∼2 meV, which corresponds to a phase difference of 0.02 radian for a fringe width of ∼20 meV (τ_1_ in the range of 100 fs). Thus, the smallest phase difference we could resolve in our experiments is as small as 0.02 radian. The differential refractive index values Δ*n* with respect to ethanol in the reference arm are indicated on the right axis of Figure 4b. The values show that the technique is extremely sensitive and is able to differentiate between very small changes in the refractive index on a scale of 10^–5^ over a broad range of frequencies. Other interferometric techniques can be more sensitive but generally are performed using selected frequencies and a coherent light source.

Though the plots shown in Figure 4a,b give a good fit with eq 1, this alone is insufficient to conclude whether the response is described purely by a complex Lorentz function or Voigt line shape or a convolution with a different line shape is needed because a similar fitting error was also observed while fitting data shown in Figure 3a with the Voigt profile. For determining the degree of inhomogeneity, we plot the response function, *t*(ω), on the complex plane in Figure 4c, which reveals that for all concentrations, the plots exhibit a nearly circular or elliptical shape with the percentage distortion values calculated to be in the range of 2–7% for the main absorption peak of solutions S1–S5. At lower concentrations, there is a perceptible deviation from circular to elliptical shape. Interestingly, there is no clear concentration dependence observed in the spectral widths obtained by fitting the Voigt profile to the spectra shown in Figure 4a. There is also a feature observed corresponding to the broad peak at 2.05 eV, but the circle is incomplete due to the limited spectral range, particularly at the higher photon energy. The difference between the change in the spectral width obtained from fitting the Voigt line shape and the phase plots shown in Figure 4c demonstrates the superiority of the proposed technique in resolving changes in inhomogeneous broadening. The absorption spectra (Figure 3a) or the transmission spectra (Figure 4a) do not provide information on whether the change in the width is due to alteration in homogeneous or inhomogeneous line widths. This is because of the limited spectral range of the measurement, the presence of overlapping spectral features, and the accuracy of line shape fitting to the convolution of Lorentzian and Gaussian (Voigt line shape) is limited. To interpret the change in the Voigt line width, suitable physical models need to be employed, which can rationalize the alterations in homogeneous and inhomogeneous broadening as a function of concentration. On the other hand, broad-band spectral interferometry provides independent information on the presence of inhomogeneous broadening. The technique is simple to implement, involves linear optical interactions, and only requires a partially coherent broad-band light source. Our results indicate that at the concentration (S1–S5) tested here, the chlorophyll solution shows increasing inhomogeneity with dilution. The changes in inhomogeneity observed at the highest and lowest concentrations are 2 and 7%, respectively. There is no significant alteration in the local environment or aggregate formation observed in these samples as a function of concentration.

**Figure 5:**
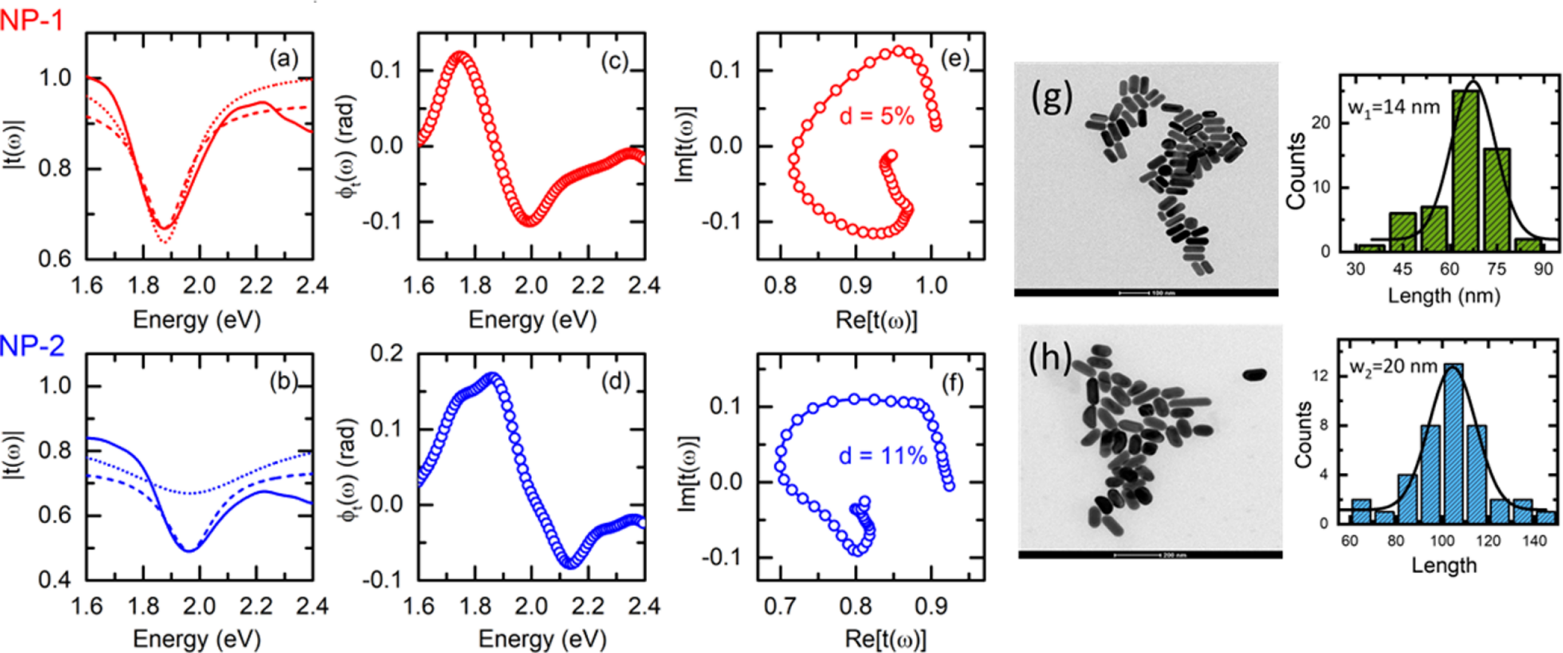
Analysis of nanoparticles NP-1 (top panels) and NP-2 (bottom panels). (a, b) and (c, d) Amplitude |*t*(ω)| and phase ϕ_t_(ω) of the response functions. In (a, b), dashed lines correspond to the Lorentz fit, whereas the dotted line corresponds to the Voigt fit. (e, f) *t*(ω) plotted on the complex plane. The values of the distortion function, *d*, suggest that NP-2 has greater inhomogeneity. TEM images shown in (g, h) verify our results. Insets depict the distribution of the length of the nanorods seen in the TEM images. They confirm wider distribution for NP-2 along with distribution of shapes, giving rise to a higher degree of inhomogeneity in NP-2.

We now test the WLSI technique to estimate the inhomogeneity of two gold nanocolloids comprising gold nanorods dispersed in water. These nanocolloids have been obtained commercially from different sources. Nanocolloid NP-1 is obtained from Sigma-Aldrich and exhibits an extinction peak due to longitudinal localized surface plasmon resonance (LSPR) at 1.88 eV and transverse LSPR at 2.39 eV. Another nanocolloid NP-2 obtained from NanoPartz, USA exhibits extinction peaks corresponding to longitudinal and transverse LSPR at 1.967 and 2.39 eV, respectively. The amplitude |*t*(ω)| and phase ϕ_*t*_(ω) of the transmission response function for the two samples are shown in Figure 5a–d. From the extinction spectra (Figure 5a,b), it is very difficult to compare the extent of inhomogeneity in these two samples simply by comparing the width of the resonances because LSPR is known to exhibit shape- and size-dependent homogeneous broadening. The response function, *t*(ω), of the two samples when plotted on the complex plane (Figure 5e,f), however, suggest higher inhomogeneity in NP-2. Percentage deviation measurements give a value of ∼5% to NP-1 and more than 11% to NP-2. In addition, the deviation of the shapes of plots from the ellipse compared to simulations in Figure 1 indicates the presence of broadening mechanisms characterized by non-Gaussian distributions. To validate our results, we performed transmission electron microscopy (TEM) imaging on the two ensembles of gold nanorods investigated here. The resulting images are shown in Figure 5g,h, respectively. The corresponding statistical analysis of the length distributions of the nanorods is shown. TEM analysis clearly verifies our conclusion that NP-2 has a greater degree of inhomogeneity than NP-1. In addition to broader length distribution, TEM images also show that NP-2 has more shape inhomogeneity compared to NP-1. This may in turn be responsible for the deviation from the Gaussian broadening and the difference between the shapes observed in Figure 5e,f. Our results once again confirm that WLSI though much simpler to implement provides information on inhomogeneity, which otherwise is possible to obtain only using high spatial resolution techniques like electron microscopy, which are significantly more expensive to implement.

## Summary

We used the WLSI technique, which involves linear optical interactions, and implemented it using a partially coherent light source to determine the complete optical response of a sample in terms of amplitude as well as phase. The technique in turn provided information about small changes (10^–5^) in the refractive index associated with the optical resonance with respect to a transparent reference over a broad spectral range. The optical response was further used to estimate the degree of inhomogeneity in a liquid sample. We also discussed experimental configurations to improve sensitivity and the S/N ratio by compensating for material dispersion and optimizing the time delay between the sample and the reference arms. The smallest phase change we accurately measured using the setup was 0.02 radian in the visible range.

The analysis of varying concentrations of chlorophyll-*a* solutions in ethanol demonstrated the robustness of the technique in estimating inhomogeneous broadening. WLSI also successfully characterized the degree of inhomogeneity in gold nanocolloids, establishing applicability to a wide range of samples. The technique based on a partially coherent broad-band source and linear optical phenomena offers a sensitive and accurate method to estimate inhomogeneity in liquids as well as colloids and can be easily extended to solid samples as well as ensembles on nanostructures. Hence, it can find applications in automated sample characterization and quality control. It can also be extended to investigate the dephasing dynamics of homogeneously broadened optical excitations in nanostructures.^44−46^
